# Chemopreventive Effects of Phytochemicals and Medicines on M1/M2 Polarized Macrophage Role in Inflammation-Related Diseases

**DOI:** 10.3390/ijms19082208

**Published:** 2018-07-28

**Authors:** Yen-Chun Koh, Guliang Yang, Ching-Shu Lai, Monthana Weerawatanakorn, Min-Hsiung Pan

**Affiliations:** 1 Institute of Food Science and Technology, National Taiwan University, Taipei 10617, Taiwan; yenkoh0123@gmail.com; 2 Hubei Key Laboratory of Economic Forest Germplasm Improvement and Resources Comprehensive Utilization, Hubei Collaborative Innovation Center for the Characteristic Resources Exploitation of Dabie Mountains, Huanggang Normal University, Huanggang 410083, China; guliangyang@163.com; 3 Department of Seafood Science, National Kaohsiung Marine University, Kaohsiung 811, Taiwan; chlai@mail.nkmu.edu.tw; 4 Faculty of Agriculture Natural Resources and Environment, Naresuan University, Phitsanulok 65000, Thailand; monthanac@nu.ac.th; 5 Department of Medical Research, China Medical University Hospital, China Medical University, Taichung 40402, Taiwan; 6 Department of Health and Nutrition Biotechnology, Asia University, Taichung 41354, Taiwan

**Keywords:** chemoprevention, phytochemicals, M1/M2 macrophage, inflammation

## Abstract

Macrophages can polarize into two different states (M1 and M2), which play contrasting roles during pathogenesis or tissue damage. M1 polarized macrophages produce pro-inflammatory cytokines and mediators resulting in inflammation, while M2 macrophages have an anti-inflammatory effect. Secretion of appropriate cytokines and chemokines from macrophages can lead to the modification of the microenvironment for bridging innate and adaptive immune responses. Increasing evidence suggests that polarized macrophages are pivotal for disease progression, and the regulation of macrophage polarization may provide a new approach in therapeutic treatment of inflammation-related diseases, including cancer, obesity and metabolic diseases, fibrosis in organs, brain damage and neuron injuries, and colorectal disease. Polarized macrophages affect the microenvironment by secreting cytokines and chemokines while cytokines or mediators that are produced by resident cells or tissues may also influence macrophages behavior. The interplay of macrophages and other cells can affect disease progression, and therefore, understanding the activation of macrophages and the interaction between polarized macrophages and disease progression is imperative prior to taking therapeutic or preventive actions. Manipulation of macrophages can be an entry point for disease improvement, but the mechanism and potential must be understood. In this review, some advanced studies regarding the role of macrophages in different diseases, potential mechanisms involved, and intervention of drugs or phytochemicals, which are effective on macrophage polarization, will be discussed.

## 1. Introduction

The mechanism of pathogens elimination by macrophage during inflammation as a response to pathogenesis or tissue damage has been clearly elucidated over several decades. Some macrophages are widely distributed throughout the body circulating in the bloodstream as monocytes and M_φ_, while other specialized M_φ_ are found in several organs and tissues, which are responsible for clearing senescent/apoptotic cells and foreign/pathogenic materials via phagocytosis, enhancing constructive processes, such as tissue repair and remodeling and wound healing [1]. M_φ_, so called M_0_ macrophages, are the macrophages that are derived from monocytes but have to undergo terminal differentiation induced by cytokines [2]. Generally, there are two major roles of M_φ_, namely regulating parts that are pivotal for the inflammatory response to disease and infection. The first essential role of resident macrophages is to produce primary pro-inflammatory cytokines and inflammatory mediators that result in inflammation, while at the same time producing anti-inflammatory cytokines and ingesting pathogens to resolve inflammation. Macrophages take part in both acute inflammatory response, as infection or tissue injury aforementioned, but also in the chronic infections, autoimmune disease. Furthermore, chronic inflammatory states are more likely to be associated with malfunction of tissue but do not directly relate to tissue repair and host defense, which widely occurs in variety of diseases, like cardiovascular diseases, obesity, and type 2 diabetes [3].

Secretion of appropriate cytokines and chemokines from M_φ_, accompanied by an array of surface receptors can lead to microenvironment modifications that are required for innate and adaptive immune responses [4]. M1/M2 polarization or transition can be controlled to ensure smooth inflammatory response for healing [5]. Spiller et al. (2015) demonstrated the effect of M1 and M2 on angiogenesis initiation and vessel maturation promotion, respectively, by modifying decellularized bone to achieve slow release of IFNγ (Interferon γ) and IL-4 (Interleukin-4) in a murine subcutaneous implantation model [6]. In fact, Li et al. (2018) introduced a three-dimensional (3D)-printed IFN-γ-loading calcium silicate-β-tricalcium phosphate scaffold, which could provide an immunomodulatory effect during significant levels of bone defect repair. Release of IFN-γ and silicon ions modulates polarization of M1 and M2 macrophages, accelerating vascularization and osteogenesis by boosting angiogenesis [7]. The importance of M2-Like microglia and monocyte/macrophage in tissue and vascular remodeling and also the infiltration of M2 into the brain by using translocator protein (TSPO) for cell-based therapies to facilitate axonal outgrowth and angiogenesis has also been reported by Kanazawa et al. (2017). The balance and distribution of M1 and M2 macrophages contribute a supportive effect in tissue regeneration, particularly M2 macrophages, which are believed to be responsible for mediating microarchitecture recovery and healing, evidenced in tendon repair [8]. In fact, the concept of ‘immune-relevant’ microbiome has also implanted, the correlations between microbiota and disease are widely discussed in a recent review [9].

M_φ_ in tissue and circulating in the bloodstream can be directed to a process that is called polarization, depending on different stimuli. Polarized macrophages can be classified into two major subtypes—pro-inflammatory M1 and anti-inflammatory M2—based on the polarization concept of type 1/type 2 helper T-cells [10]. After classically activated macrophages (CAM, so-called M1 macrophages) were first recognized to be activated in transformation by IFN-γ, the main product of Th2 (type 2 helper T-cell), IL-4, was found to convert macrophages into M2 macrophages. It is clearer in directing or manipulating the reprogramming of monocytes/polarized macrophages into two major polarization states by stimuli exposure, generally IFN-γ, lipopolysaccharid (LPS), or TNF (Tumor necrosis factor) and GM-CSF (Granulocyte-macrophage colony-stimulating factor) for M1 activation while IL-4 and IL-13 for M2 polarization directing [11]. Adam et al. (2014) described nearly all the states of polarized macrophages, including the subtypes M2a, M2b, M2c, and M2d, together with M4, Mhem, and Mox, which are still not sufficiently recognized. Some of the major surface markers of different states of polarized macrophages and cytokines can alternatively activate the transformation of M_φ_. After polarization, the phenotypes of different polarized macrophages produce specific chemokines in significant concentrations, which can be used as indicators of transformation. Chemokines play crucial roles in inflammatory reactions and chemokine receptors were found to express differentially on polarized Th cells. Typically, chemokines produced by M1 macrophages include CCL5 (Chemokine (C–C motif) ligand 5), CXCL9 (Chemokine (C–X–C motif) ligand 9), 10, 11, and 16, while M2 macrophages secret differently, CCL17, 18, 22 and 24 by M2a, CCL1 by M2b and CCL13, 16 and 18 by M2c [12]. On the other hand, polarization differentially affects the progression of diseases, including atherosclerosis, obesity and insulin resistance, tumor progression, and bone destruction [4,10,13,14]. The transformation of M_φ_ into pro-inflammatory M1 leads to significant amounts of proinflammatory cytokines and chemokines production; thus, M1 are typically associated with inflammation, related to tissue damage and tumoricidal activity, while activated macrophages (M2) are normally characterized by the production of anti-inflammatory cytokines. Therefore, the involvement of M_φ_ in the progression of different diseases is no longer representative of different states of polarized macrophages during therapy, since they play different roles after transformation. Nonetheless, macrophages represent highly suitable targets for anti-inflammatory therapies. The inducers and samples intervened in the studies mentioned in this review, which may lead to M1 and M2 polarizations (with different isolated macrophages and/or macrophage cell lines, and macrophages in different tissues), are listed in Table 1 and Table 2.

In this review, the potential involvement and specific roles of polarized macrophages in several diseases are underlined, and the intervention of phytochemicals as preventive or therapeutic strategies focusing on directing transformation of M_φ_ into M1/M2 macrophages will be discussed. The focus is primarily placed on research between 2014 to 2018.

## 2. Preventive and Therapeutic Strategies Dealing with M1/M2 Macrophages Polarization

As previously mentioned, the inflammatory response normally occurs during tissue damage or infection as a protective mechanism to remove stimuli, but an uncontrolled inflammatory response may lead to severe outcomes due to the dysfunction of the tissue environment. Fortunately, determining the role of M_φ_ transformation has become an important step that is related to M1 macrophages being more likely to be characterized to produce high levels of pro-inflammatory cytokines, with M2 macrophages expressing anti-inflammatory effectors. Therefore, the polarization of macrophages to transform into either M1 or M2 phenotype has become an attractive solution to deal with inflammation-associated diseases. The most common strategies for inflammation resolution dealing with M_φ_ transformation is by promoting M2 and inhibiting M1 macrophage polarization, with the latter being more frequently used. The members of peroxisome proliferator-activated receptor (PPAR), interferon regulatory factor (IRF), signal transducers and activators of transcription (STAT), hypoxia inducible factor (HIF), nuclear factor-κB (NF-κB), and Kruppel-like transcription factor (KLF) families are now recognized as the major determinants of M1 and M2 polarization [51]. The following describes some advances in recent studies that are related to M1 and M2 polarization, along with the efficacy of some phytochemicals and nutraceuticals after intervention.

**Figure 1:**
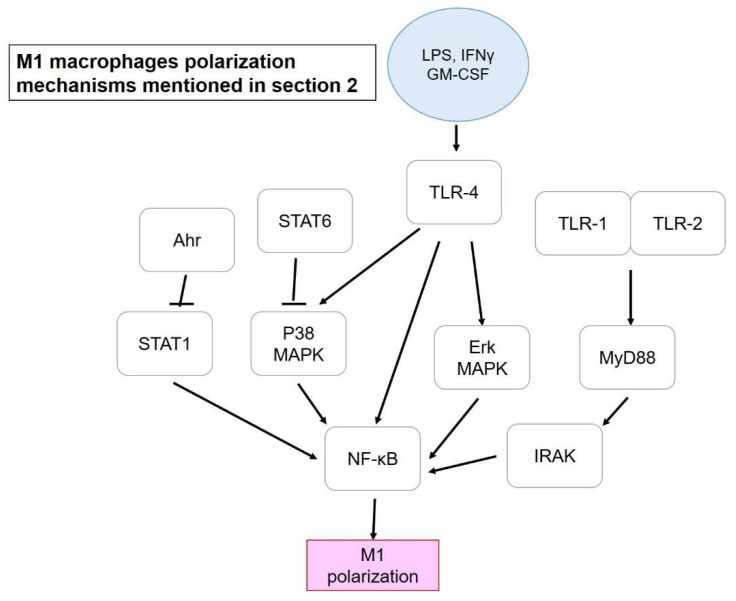
M1 macrophage polarization mechanisms mentioned in Section 2. Blue circle, inducers; white blocks, genes/proteins involved.

First are provided examples that are related to inhibiting M1 polarization rather than promoting M2. Potential mechanisms modulating M1 polarization are as presented in Figure 1. Bone-marrow derived macrophages (BMDM) were stimulated for polarization into M1 and M2 macrophages in the study of Xu et al. (2016) by using IFN-γ and IL-4, respectively. However, M1-macrophage marker CD11c and iNOS (Inducible nitric oxide synthase) and the phagocytosis activity of M1 (by calculating latex bead ingestion rate) had dose-dependently decreased after intervention with isomeranzin extracted from *Murraya exotica.* In contrast, M2 markers, such as *Arg1* (Arginase 1), *Fizz1* (Retnla resistin like alpha), and *Ym1* (Chil3 chitinase-like 3) showed no difference after isomeranzin treatment, indicating no significant impact on M2 polarization. Isomeranzin also showed an inhibitory effect on NF-κB (nuclear factor kappa-light-chain-enhancer of activated B cells) activation via acting as an antagonist of TLR4 (Toll-like receptor 4) and blocking ubiquitination of TRAF6 (TNF receptor-associated factor 6), respectively. Furthermore, isomeranzin exhibited a suppressive action on M1-associated inflammation in Lipopolysaccharide (LPS)-induced sepsis, and dextran sulfate sodium (DSS)-mediated and 2, 4, 6-trinitrobenzne sulphonic acid (TNBS)-stimulated colitis in a mouse model. Significant reduction in both M1-associated protein level and mRNA expression of *IL*-*1β*, *IL*-*6*, *TNFα,* and *iNOS*, and the attenuation by isomeranzin has been reported, possibly via reducing phosphorylation of ERK and p65 [37]. Another study by Feng et al. (2014) suggested that pentamethoxyflavone (PMFA) could regulate macrophage activation and ameliorate sepsis. Reduction in M1 markers and enhancement of M2 polarization accompanied of a phenotype shift from M1 to M2 was demonstrated after PMFA treatment. STAT1 (Signal transducer and activator of transcription 1) inhibition and STAT6 activation were found to be responsible for M1 repression and M2 induction, respectively, concomitant with increased expression of downstream protein expression of STAT6, including SOCS1 (Suppressor of cytokine signaling 1) and inhibition of NF-κB and MAPK (Mitogen-activated protein kinase) signaling pathways. IL-4 (M2 inducer) was found to activate type I and type II receptors, with the former facilitating phosphorylation of JAK3 (Janus kinase 3) while JAK1 by latter, and both resulting in STAT6 phosphorylation. Besides, activation of the former also activates IRS-2 and PI3K/Akt pathways, which was enhanced by IGF-1, and which led to M2 polarization. Another M2 inducer (IL-13) could also activate type II IL-4 receptor but it could not upregulate the expression of M2-related markers, such as IL-4 [52]. Finally, protection from sepsis by PMFA was achieved as via inhibition of M1 polarization. Injection of PMFA pretreated-M1-BMDM but not M2-BMDM also improved the survival rate of mice suffering from sepsis, which indicated the importance of the presence of M1 macrophages in treatment as long as the M1/M2 ratio was under appropriate balance [22].

**Figure 2:**
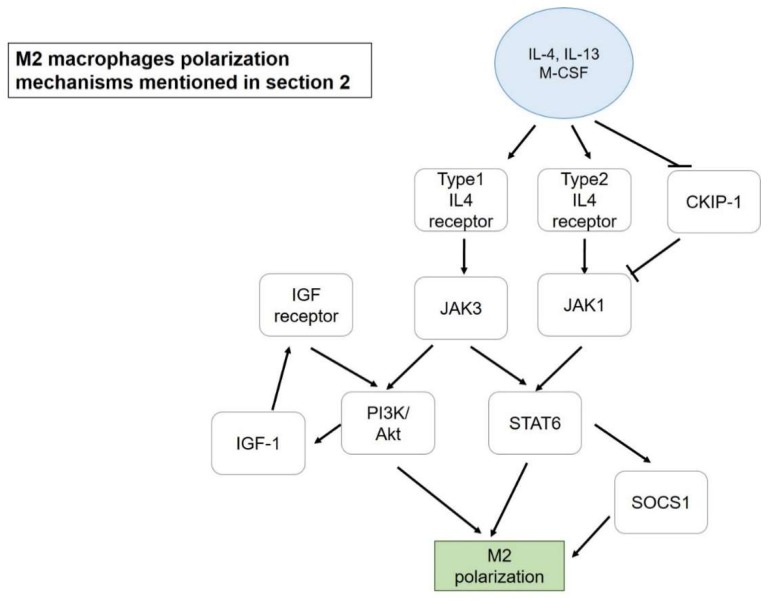
M2 macrophage polarization mechanisms mentioned in Section 2. Blue circle, inducers; white blocks, genes/proteins involved.

The aforementioned promotion of M2 could be another strategy, rather than inhibiting M1, to resolve inflammation, and the mechanisms that are mediating M2 polarization mentioned in this review are presented in Figure 2. Among 11 ginsenosides, Rg_3_ showed a positive impact on M2 polarization. After being treated with LPS, isolated mouse peritoneal macrophages dramatically expressed several M1 marker genes, such as *COX*-*2* (Cyclooxygenase), *iNOS*, *IL*-*1β,* and *TNFα*. Pretreatment with Rg_3_ successfully restored a representative M2 marker (arginase-1) which had reduced after treating with LPS. To further investigate the protective effect of M2 macrophages to the peritoneal cavity, a zymosan-induced peritonitis mice model was used. It was suggested that M2 macrophages contributed a protective advantage to accelerate the removal of polymorph nuclear lymphocytes, including neutrophils, eosinophils, and basophils that are normally found in the early stage of peritonitis after stimulation [27]. Besides ginsenoside Rg_3_, curcumin also showed a significant effect on M2 macrophage promotion. IL-4 and IL-13 secretion was induced by curcumin in both mRNA and protein level, and the up-regulation of these cytokines led to M2 macrophage polarization. IL-4 and IL-13 could activate STAT6 via type-1 and -2 IL-4 receptors, followed by the STAT6-dependent pathway for M2 polarization. The effect of curcumin was further determined in an experimental antoimmune myocarditis (EAM) heart injury rat model, with curcumin improving M1 macrophage infiltration and transformation of M_φ_ and M1 into M2 macrophages [24]. On the other hand, a combination of phytochemical extracts from the African-origin plant *Sutherlandia frutescens* demonstrated its potential to transform M1 (LPS-stimulated Raw264.7) into M2a macrophages. Ethanolic extract significantly reduced M1 surface marker CD86 and in parallel increased CD206 expression (M2 marker), indicating the redirection from M1 to M2 polarization. Furthermore, NF-κB activation tended to be repressed after treatment via the down regulation of ERK ½ and p38 MAPK signaling pathways, starting from inhibition at TLR-4 [16]. Similar to TLR-4, a study of M1 polarization mediated by OmpU, an outer membrane porin protein of *Vibrio cholera*, reported that TLR-1/TLR-2 heterodimers could lead to recruitment of MyD88 (Myeloid differentiation primary response 88) onto the complex, followed by IRAK (Interleukin-1 receptor-associated kinase 1), which led to translocation of NF-κB and M1 polarization with the production of TNFα and IL-6 [53].

Alteration of M1/M2 macrophage polarization can be crucial to regulate the immune response. Therefore, many studies have focused on clarifying the mechanism of regulating macrophage polarization. For instance, Climaco-Arvizu et al. (2016) discussed the importance of aryl hydrocarbon receptor (Ahr) in the transformation of macrophages into M1/M2. Ahr was suggested to have a suppressive effect on NF-κB activation via interaction with STAT1. After differentiation into M1, Ahr-null peritoneal macrophages in M1 state led to higher expression of M1-associated marker gene compared to wild type macrophages. This result indicated the negative correlation of Ahr level to proinflammatory M1 phenotypic expression via activation of NF-κB. However, iNOS expression, which was positively regulated by Ahr, decreased in Ahr-null cell and led to lower NO production and weakened phagocytic capacity due to lower bacteria killing being essential without ROS production enhancement. Moreover, M2 markers were almost undetectable in Ahr-null cells after being polarized with IL-4, particularly IL-10, and lacking IL-10 might lead to Th2 immunity development due to inhibition of proinflammation cytokine production from T cells [19]. Therefore, the importance of Ahr was pointed out in macrophage transformation into M1/M2 macrophages in this study. Besides Ahr, Casein Kinase 2 Interacting Protein 1 (CKIP-1) was suggested as another molecule that could manipulate macrophage speciation. CKIP-1 expression was positively correlated to M1 stimuli (LPS and IFN-γ), but being suppressed by M2 stimuli (IL-4 and IL-13). Therefore, inhibition of CKIP-1 could be another strategy to avoid its suppressive effect on the M2 polarization pathway and the JAK1-STAT6 signaling pathway. Lastly, the inhibition of CKIP-1 was suggested as an effective treatment for acute inflammation induced by LPS-induced sepsis and 12-*O*-tetradecanoylphorbol-13-acetate (TPA)-induced cutaneous disease [54].

Focusing on specific proteins involved in polarization, energy producing mechanisms can be another target to be modulated. The importance of glycolysis in cytokine production of M2 macrophage regulation was proposed by Chiba et al. (2017). After treating polarized macrophages with LPS, significant increment in glucose-6-phosphate and glycolysis-related metabolite concentrations in medium of M2 but not in M1 macrophage incubation was observed. The involvement of glycolysis as intracellular metabolic pathways of macrophages after being induced by LPS was further confirmed by 2-deoxy-d-glucose (2-DG) and dichloroacetate (DCA), the inhibitors of glycolytic and anaerobic glycolysis pathways. Production and mRNA expression of IL-10 were found to be significantly reduced by 2-DG but not DCA. Furthermore, a reduction in ERK phosphorylation indicated that ERK could play a key role in the production of cytokines, such as IL-10. On the other hand, 2-DG also was found to inhibit IL-6 production via binding to the C/EPBβ promoter, showing that not only IL-10, but also other cytokines’ productions might also be regulated by glycolysis [18]. It seems that the cellular metabolism plays an important role in cellular signaling for cytokine production. Therefore, this study provides the concept to modulate the function of macrophages by regulating macrophage intracellular metabolism.

In the following sections, the role of macrophages at different states in various diseases or disorders, including liver injuries, cancers, obesity and metabolic syndromes, colon health, and brain damage, and neuron injury will therefore be discussed, accompanied by the intervention of phytochemicals or drugs suggested to play roles in disease therapy or prevention.

## 3. Role of Macrophages in Intestinal and Colorectal Disease

**Figure 3:**
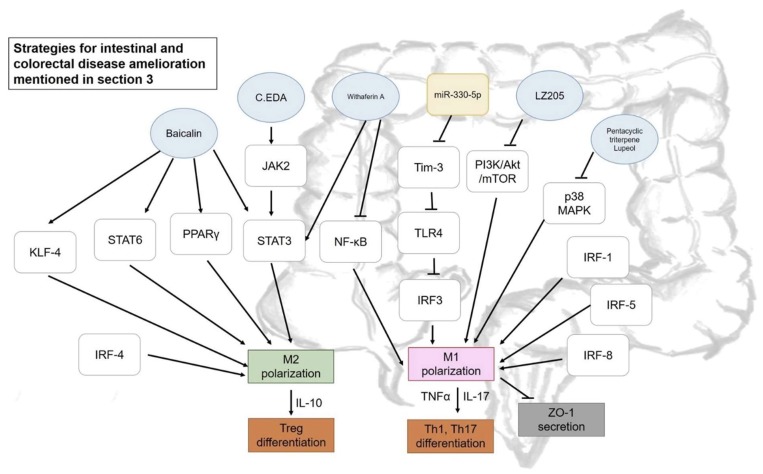
Some strategies proposed for intestinal and colorectal diseases amelioration mentioned in Section 3. Blue circle, samples intervened; yellow block, microRNA intervened; white blocks, genes/proteins involved.

TNBS- and DSS-induced colitis models are widely used to discuss anti-inflammatory issues. The role of M1/M2 macrophages in inflammatory bowel disease will be further discussed in this section, along with some potential ameliorative actions by phytochemicals that have been reported in recent years, as presented in Figure 3. Interestingly, mesenchymal stem cells (MSCs) were recently highlighted due to their immunomodulatory properties and the regulatory effect of adipose tissue-derived MSCs (ASCs) was reported in colitis amelioration. MSCs were reported to release anti-inflammatory cytokines when being stimulated by inflammatory factors. Co-culturing THP-1 and ASCs led to the expression of M2-related cytokines and chemokines, including CD206, CD68, CCL18, legumain, and IL-10, concomitant with an inhibitory effect on IL-18 secretion and formation of inflammasome complex (ASC/Cas-1/NLRP3) due to significant increment in PGE2 production. This result was confirmed in a DSS-induced chronic colitis model with reduction in the number of polarized M1, indicating the potential of ASCs in suppressing the inflammatory response and its potential for treatment of inflammatory bowel disease [55].

Some pharmaceuticals were suggested as having effects on macrophage polarization to ameliorate inflammation-related diseases. Zhang et al. (2017) demonstrated an improvement in DSS-induced colitis by promoting M2 macrophage polarization by combining (+)-borneol and edaravone (EDA), so-called C.EDA. The level of M1-associated cytokines dramatically decreased in the serum of DSS-induced mice after treatment, and M1 macrophage infiltration sharply decreased concomitantly with an increment in M2 macrophages and Caudin-1 protein level. Activation of M2 polarization by C.EDA could be achieved by promoting JAK2 that led to STAT3 translocation and activates M2-related cytokines [39]. Phosphorylated STAT3 up-regulation and the inhibition of NF-κB were reported to be essential for macrophage repolarization and switching M1 into M2 phenotype, a result that was confirmed by using withaferin-A (steroidal lactone purified from ginseng or Indian winter cherry) [56]. Further, immune regulators could also be targeted to achieve M1/M2 regulation in disease attenuation. Tim-3 (T cell Ig mucin-3) is typically associated with its function in negatively regulating T cell response. However, Tim-3 could also be considered as the regulator of innate immune cells, which include macrophages and their polarization process. Tim-3 was found to lead to down-regulation or blockage associated with weight loss and tissue damage with an enhancement of M1 macrophage level and reduction in M2-related expressions. This result was further confirmed by the adoptive transfer of Tim-3-silenced macrophages and transgenic Tim-3 overexpression mice, which resulted in exacerbated colitis and inhibition of M1 response, respectively, indicating the inhibitory effect of Tim-3 in M1 polarization. The mechanism was further confirmed that the inhibitory effect of Tim-3 was via inhibiting the TLR4-IRF3 pathway [26]. Nine IRFs have been identified, with three of them (IRF-1, IRF-5, and IRF-8) being normally associated with the polarization mechanism of M1 macrophages, while IRF-3 and -4 are more often found to be involved in M2 polarization [57]. Some studies support that microRNAs also widely take part in M1/M2 polarization. Sun et al. (2018) revealed that miR-330-5p level increased in obese mice, which consequently inhibited Tim-3 while the inhibition of miR-330-5p could successfully enhance Tim-3 expression, leading to M2 polarization and attenuated insulin resistance [58].

Some phytochemicals also exhibit the potential for disease improvement via multi-target therapeutic strategies, with anti-inflammatory effects forming a particular focus. For instance, baicalin, a flavonoid isolated from *Scutellaria baicalensis* Georgi, led to a significant decrease in the M1/M2 macrophage ratio in LPS-induced mouse peritoneal macrophages, indicating deviating polarization from M1 to M2 subsets, particularly the M2a subtype with *Fizz1* expression. A similar result was obtained in lamina propria mononuclear cells that were isolated from a DSS-induced colitis mice model. The IRF signaling pathway, along with several transcription factor, such as STAT3, STAT6, PPARγ, and KLF-4, were reported to promote the M2 macrophage phenotype. Up-regulation of IRF4 and down-regulation of IRF5 after the addition of baicalin consolidated the result of M2 polarization [42]. A similar finding was obtained by Zhu et al. (2016), who reported alleviated inflammatory bowel disease by switching macrophages (CD14^+^ monocytes isolated peripheral blood mononuclear cells) from M1 state to M2 by intervention of the pentacyclic triterpene Lupeol. The transformation of macrophages was evidenced by a change of reduction in the CD86 M1 marker and increment in the CD206 M2 marker. IRF-5 protein, which promotes M1 polarization, was significantly reduced via inhibition of p38 MAPK phosphorylation. It was suggested that soluble factors that are released by M1 macrophages were responsible for disruption of epithelial integrity by reducing ZO-1 (Zonula occludens-1) expression, with the effect being reversed by Lupeol [41].

The TNBS-induced colitis murine model was also used by Jang et al. (2017) to determine the anti-inflammatory effect of 4-methocylonchacarpin, an 80% ethanol extract of *Abrus precatorius* Linne. TNBS intra-rectal administration resulted in an increment of M1 polarization markers, including TNFα, arginase-2, and IL-1β expression and immunohistochemical examination revealed the infiltration of activated M1 macrophages with the CD86 surface marker. Increased levels of TNFα and IL-17 may indicate the differentiation of Th1 and Th17 accompanied with M1 polarization. The intervention of 4-methocylonchacarpin inhibited Th1 and Th17 differentiation with a significant reduction in IL-17 and TNF after treatment. Furthermore, after being treated with sample, IL-10 (M2 marker) that may stimulate Treg differentiation, significantly increased [25]. On the other hand, the down-regulation of PI3K/Akt/mTOR signaling pathways by a newly synthesized flavonoid, LZ205, has been reported as a critical strategy to inhibit M1 polarization in both LPS-stimulated Raw 264.7 murine macrophages and DSS-induced colitis and Alum-induced peritonitis mice models [15].

In brief, M1 brings significantly negative effects in colon inflammatory disease and M2 induction becomes a particularly important strategy for amelioration. Nonetheless, the mechanisms of M1 and M2 polarization have to be further illustrated to determine their potential for disease prevention and improvements.

## 4. Role of Macrophages in Liver Protection

The role of polarized M1 and M2 macrophages may be similar to other diseases, with the inhibition of pro-inflammatory M1 and the promotion of anti-inflammatory M2 macrophages being a desired strategy. For instance, inflammatory TNFα is suggested as limiting systemic insulin action, which may combat metabolism throughout the body [59]. Nonetheless, one of the exceptions will be discussed in this section.

The roles of polarized macrophages was mentioned in a study of a Trioacetamide (TAA)-induced hepatic cirrhosis rat model, which found significant numbers of CD68^+^ macrophages in the clusters or pseudolobules of adipophilin (Adp)-positive hepatocytes. Adipophilin is a widely-used marker of lipid accumulation in hepatocytes during the occurrence of centrilobular necrosis induced by TAA. The number of Adp-positive pseudolobules significantly increased along the cirrhosis progression and the expression of adipophilin affected the polarization of different M1 and M2 macrophages. CD68^+^ M1 macrophages significantly increased in Adp-negative pseudolobules, while M1 with Iba1 expression and CD163^+^, CD206^+^, and gal3-positive M2 sharply increased in Adp-positive pseudolobules. In addition to this, M1- and M2-related factors, including TNFα, MPC-1, and Iba1 from M1 macrophages and IL-4, TGF-β, and Gal-3 from M2 macrophages increased accordingly. It has been suggested that macrophage infiltration is strongly correlated to fatty deposition development, with adipohilin expression being used as an indicator. Since M2 macrophages were activated in the later stage to ameliorate liver injury, the polarization of M1/M2 macrophages can be used as an indicator of steatosis and may be an entry point for liver protection [46]. Alcoholic hepatitis is a life-threatening condition related to inflammation among alcoholic liver patients. It had been pointed out that chronic ethanol abuse could lead to M1 polarization of Kupffer cells due to repressed expression of miR125a, let-7c, miR221, and miR222 of polarized M2, and the repressive effect could be reversed by hyaluronic acid that decreased sensitivity of Kuffer cells to LPS. A similar effect was confirmed in peripheral blood mononuclear cells in alcoholic hepatitis patients [60].

To clarify the mechanism of macrophage polarization, Han et al. (2017) demonstrated liver macrophage transformation via the RORα mediated signaling pathway and revealed that M2 could provide protection against nonalcoholic steatohepatitis (NASH). Kuffer cells that were isolated from mouse liver switched into CD206^+^ after being induced by IL-4 with a concomitant increment in RORα level. The involvement of RORα (RAR-related orphan receptor alpha) was confirmed by using agonist, inverse agonist, RORα ligand (JC1-40), and shRORα. A similar finding was also obtained in transformation of bone-marrow-derived macrophages. Furthermore, KLF4 (Kruppel-like factor 4), a major M2 polarity switch regulator, was evidenced as a downstream gene of RORα. In an animal model, HFD-fed LysM^Cre^-RORα^fl/fl^ illustrated a lower level of M2 markers in RORα-deficient KCs, and resulted in severe hepatic steatosis, increment in serum glutamic pyruvic transaminase (GPT) and glutamic oxaloacetate transaminase (GOT), and hepatocyte ballooning with lipid droplets. Communication of Kuffer cells and hepatocytes could be disrupted during RORα-deficiency that stopped M2 polarization of Kuffer cells, and IL-10 secreted by M2 was found to be compulsory in the cross-talk. Lastly, RORα expression was found in low concentrations in human hepatitis patients, which is in accordance with the findings of this study [32].

In most studies, M2 showed ameliorative effects in most diseases rather than M1 or M_φ_ macrophages, but there are some exceptions. It is understood that wound healing can be promoted by the overexpression of anti-inflammatory molecules by enhancing fibrosis that limiting further inflammatory condition [61]. However, fibrosis, one of the wound healing mechanism promoted by M2 macrophages may lead to unintended consequences in some diseases progression. One of the examples was found in liver fibrosis, which revealed that M1 but not M2 macrophages ameliorated liver fibrosis in a CCl_4_-induced hepatic fibrosis mouse model. CCl_4_ were injected twice a week for 12 weeks and mice were infused with polarized M_φ_, M1, and M2 macrophages at the eighth week. Surprisingly, not only did the M_φ_ group show a significant reduction in extracellular matrix deposition, but decrements in collagen1α1 mRNA expression and the hydroxyproline (HYP) level were also observed in the M1 group. Moreover, collagen degradation was confirmed with increased HYP in urine after M1 infusion. Besides, the up-regulation in anti-fibrotic matrix metalloproteinases (including MMP-2, -9, and -13) and the down-regulation of liver fibrosis marker, tissue inhibitor of metalloproteinases (TIMPs), were detected in both M_φ_ and M1 infusion groups. It was suggested that MMP-2, -9 and -13 might be highly related to MMP-12 or IL-13 expression, although MMP-12 and IL-13 may exhibit contrasting roles in fibrosis [62]. Similar therapeutic effects were obtained in another designed experiment that infused polarized M1 macrophages at the 16th week CCl_4_ injection and also BDL-induced hepatic fibrosis. Nonetheless, there was no significant effect that was obtained in all M2 infusion groups in different designs. A remarkable reduction in α-SMA (alpha-smooth muscle actin) and desmin indicated a decrement in the activated number of hepatic stellate cells (HSCs), and TUNEL-positive marker accompanied with an increased number of NK cells suggested that the decrement was due to apoptosis of HSCs (but not hepatocytes) after tail vein infusion of M_φ_ and M1 polarized macrophages. Another novel finding of this study was a new classification of restorative macrophage with Ly6c expression that was recruited by M1 and M_φ_, which is responsible for anti-fibrosis and hepatocyte proliferation improvement effects [33]. It has been reported that polarized macrophages also play important roles in fibrosis of different organs including kidney and cardiovascular systems, suggesting the importance of maintaining the balance of M1/M2 polarization to avoid the occurrence of pro-fibrotic events [63,64,65]. The circumstance suggested promoting neither M1 nor M2 macrophage polarization in disease amelioration was in development of glutathione S-transferase-placenta form-positive (preneoplasetic) lesions in a TAA-induced liver cirrhosis rat model. Notably, both M1 and M2 macrophage expression were positively correlated to development of GST-P-positive preneoplastic lesion and M2-related cytokines might have a suppressive effect on MHC class II-positive macrophages which possesses anti-tumor effects [66]. Therefore, both M1 and M2 play deleterious roles in disease development and the overexpression of M1- and M2-related cytokines should be retuned prior to further therapeutic action.

In summary, not only M2, but also M1 and M_φ_ play important roles in liver disease improvement or therapy. Microenvironments mediated by macrophages in each organ with different issues may bring unexpected results, and therefore the role of polarized macrophages must be sufficiently clarified.

## 5. Role of Macrophages in Brain Injuries and Neuron Protection

Microglia are the brain-resident immune cells in the central nervous system (CNS) and similar to most situations, M1 and M2 macrophages are the major activated phenotypes after polarization. The markers of M1 and M2 polarized microglia in human glioblastoma and surrounding normal parenchyma for therapeutic use are iNOS and CD163 for M1 and Arg-1 for M2, although there was no significant correlation that was reported between these markers and patient survival time [67]. In some brain diseases, M1 macrophage polarization is activated rapidly, may be right after 6 h during the acute phase, and normally induces neuronal toxicity, tissue damage, demyelination, or even neuronal death with a large amount of pro-inflammatory cytokines released, with M2 gradually increasing until reaching the peak of disease and functionally promoting phagocytosis, Th2 and regulatory T cell differentiation, dampening Th1 cell activity, and accelerating tissue repair, majorly for long term recovery from 1 day to 14 days or longer [68]. For instance, it was proposed by Mi et al. (2018) that shifting of M1 microglial from M2 occurred when developing stress-induced hypertension in a rat model, although M2 markers were found to be higher during the early stage of disease development [69]. Therefore, enhancing M2 polarization of microglia or developing new drugs that can promote M2 polarization may provide highly appropriate targeted therapies in CNS diseases, such as stroke, Alzheimer’s disease, and Parkinson’s disease [70,71]. Chen et al. (2017) noted that advanced glycation end products (AGEs) could lead to non-specific neuroinflammation via the RAGE/Rho/ROCK pathway and the inhibition of RAGE/ROCK not only avoids polarization of pro-inflammatory macrophages (M1) but also surprisingly promoted shifting of M1 phenotype to M2 in BV cells [72]. It was suggested that M2 is more capable to induce phagocytosis to avoid secondary inflammatory responses and promote tissue regeneration, and the selective activation of M2 polarization may be highly related to CREB (cAMP response element binding protein) expression, which is similar to the signification of NF-κB to M1 polarization [73]. Although the increment in M1 phenotype is a typical result in acute brain injuries that has also been reviewed by Zhang et al. (2017) and Lan et al. (2017) mentioned about the M1 polarization after intracerebral hemorrhage (ICH), the phenomenon is still not solid because analysis time-points were not the same [74,75,76].

Peng et al. (2017) demonstrated changes in M1 and M2 phenotypic expression by using an alcohol exposure rat model because alcohol use disorder is considered as one of the neurodegenerative conditions. CD11b^+^ myeloid cells were isolated from hippocampus and entorhinal cortex after different alcohol exposure periods. The frequency of CD11b (CD11b expression increased with microglia activation) significantly increased after a four-day binge alcohol exposure, and activation of microglia was further evidenced with increasing expression of CD45, with CD45^high^ microglia being described as activated microglia/macrophages involved in CNS injury. MHC II (M1 marker) was identified in both CD45^low^ (microglia) and CD45^high^ (activated CNS macrophage) cells, higher on the second day after a 4-day alcohol gavage. The original expression of MHC II was higher in CD45^high^ macrophages compared to nearly negative in microglia, but alcohol exposure did not increase expression of MHC II in CD45^high^ cells. Similar results were obtained in CD206^+^ cells, although CD206 is known as an M2 marker. Moreover, CD86 and CD32 (M1 markers) were also higher in CD45^high^ macrophages, but in contrast, both CD86 and CD32 expression did increase significantly after alcohol exposure in the CD45^high^ population in the entorhinal cortex. Therefore, the study illustrated the increment of M1 macrophages after alcohol exposure, as evidence of AUD induction in a mice model. Meanwhile, increment in M2 polarization was expected as a reparative response in an AUD model [49].

M1 and M2 responses are also found in traumatic brain injury (TBI), which is a leading cause of neurological disorder, which is responsible for secondary injury cascade and recovery processes. The time course of each cytokine in the hippocampus was analyzed within 24 h post injury in a CCl-induced head trauma rat model. The study explained the importance of the combination of M1 and M2 responses, instead of M1 inhibitory action and centralized in M2 promotion in most therapeutic strategies. Indeed, rapid M1 response may assist in macrophage polarization, including M2 phenotype up-regulation to neutralize the pro-inflammatory effect of M1 macrophages soon after injury, and M2 polarization was suggested to be initiated earlier for recovery enhancement [43]. Similar results were obtained in the study of Kumar et al. (2016), which further suggested that NADPH oxidase (NOX2) was responsible in M1 but not M2 polarization by using an NOX2-knockout mice model. NOX2 deficiency not only activated M2-like cells via IL-4Rα signaling, but also limited tissue loss, avoided neurodegeneration, and promoted recovery in CCl-induced cortical injury [77].

Drugs were also found to be related to M1/M2 polarization, which may bring unexpected effects to their original therapeutic action in some clinical studies. Sevoflurane, which is a commonly used volatile anesthetic, causes cognitive impairment and neuroinflammation by enhancing transcription activity of NF-κB and consequently increasing IL-6 level, which was highly related to M1 polarization. In the study of Pei, Wang, and Li (2017), sevoflurane showed a suppressive effect on M2 transformation from microglia with decrement in M2 markers, Arg1, Ym1 and IL-10 protein levels. On the other hand, due to a previous study suggesting that human umbilical cord mesenchymal stromal cells (HUC-MSC) could polarize macrophages and lead to the transformation of M1 into M2 phenotype (also confirmed in the study of Pei et al.), the authors determined the effect of sevoflurane on HUC-MSC-stimulated M2 polarization. Unsurprisingly, M2 markers reduced significantly in HUC-MSC medium-treated microglia and IL-4 induced primary microglia cells after pretreating with 2% and 4% sevoflurane. Moreover, the suppression of M2 polarization was due to changes in IL-4 signaling, subsequently leading to repressive effects on STAT6 phosphorylation and suppressing cytokine signaling protein 1 (SOC1) expression, and STAT6 could no longer down-regulate levels of SOC3, which finally resulted in the blockage of M2 polarization [31]. Fluoxetine and S-citalopram are selective serotonin reuptake inhibitors (SSRIs) that are used for depression treatment. Some antidepressants can normalize serum pro-inflammatory cytokines that may be related to depression and inhibiting the activation of peripheral immune cells. Based on that, Su et al. (2015) demonstrated the impacts of these SSRIs on M1/M2 polarization to determine other potential therapeutic options. Typical results were obtained after the intervention of SSRIs that M1 polarization was inhibited, while M2 transformation was enhanced in both BV2 and primary microglia cells. Nonetheless, it was further suggested that the effect of SSRIs, particularly fluoxetine previously noted in another study, may promote M1 polarization at lower concentrations while providing an inhibitory effect at higher concentration, and the effect of these antidepressants might be the opposite for activated and resting microglia cells [36]. Association of demyelination of CNS to neurodegenerative disorders has been previously reported and progesterone is known as a myelin expression generator in CNS. Aryanpour et al. (2017) illustrated the significant suppressive effect on M1-associated receptors and enzymes in cuprizone-induced demyelination mice after progesterone therapy, which is concomitant with the promotion of M2 polarization. Inflammasomes are responsible for activation, maturation, and secretion of cytokines. For instance, NLRP3 (NACHT, LRR, and PYD domains-containing protein 3) can activate secretion of IL-1β and IL-18. NLRP3 and IL-18 mRNA expression dramatically increased after cuprizone treatment and the adverse effect was reversed after progesterone therapy. Therefore, progesterone was concluded as having ameliorative effects to induce remyelination [44]. Some studies have determined the intervention of agonist or antagonist in disease amelioration. For instance, it was reported that cannabinoid receptor-2 (CB2R) could mitigate brain injury and microglial accumulation, accompanied with the conversion of M1 to M2 macrophages, and the effect could be elevated with JWH133, an agonist of CB2R via the cAMP/PKA pathway that enhanced proliferation, migration, and phagocytosis of macrophages with M2 phenotype [78]. These results provide some new ideas regarding CNS attenuation, and hopefully, some natural compounds possessing similar effects as JWH133 will be discovered in future research. Cerebral edema is a life-threatening condition that can lead to intracranial hypertension due to an accumulation of fluid in the brain, and hypertonic saline and mannitol are usually used to remove free water through the concept of osmotic force and reducing peripheral vascular resistance. In a recent study from Wen et al. (2018), hypertonic saline was also found to be effectual in microglial polarization regulation in cerebral edema treatment via the miR-200b/KLF4 signaling pathway. Similar to miR-330-5p that down-regulates Tim-3, miR-200b expression was induced in cerebral edema, concomitant with M1 polarization and reduction in KLF4 level. Hypertonic saline could significantly decrease miR-200b expression and induce KLF4, which the latter might cooperate with STAT6 to induce M2 polarization system and reduce M1 via the inhibition of NF-κB activation [79].

Besides pharmaceuticals, some phytochemicals also exhibit significant effects on macrophage polarization. Saponin diosgenin glucoside (Dios), an extract from *Tritulus terrestris* L., showed a compelling protective effect on neurons among 13 saponin extracts. Not only did it prevent activity-induced cell death induced by LPS, but Dios suppressed M1 in both mRNA expression and protein level but showed effectual action on neither mRNA nor protein expression of M2 markers. Phosphorylation of IκB-α, p38 MAPK and ERK MAPK pathways were markedly repressed by Dios, and therefore, the polarization of M1 was inhibited. Lastly, the neuroprotective effect of Dios was determined. The effect of activated microglia on neurons is mostly due to soluble neurotoxic factors released but the condition medium of LPS-activated microglia showed no negative effect on Neuro-2a cells with addition of Dios, indicating that the indirect protection of Dios to neurons may be due to reduction in pro-inflammatory molecules [38]. This study provides additional options rather than inhibiting M1 polarization alone to prevent or deal with neurodegenerative disorders. On the other hand, Meireles et al. (2016) illustrated the effect of anthocyanin, including cyanidin, cyanidin-3-glucose and mixture of 3′- and 4′-methylcyanidin-3-glucose on the M1/M2 phenotype of microglia. There was no significant impact on microglia phenotype without any stimulation, but after being induced by LPS, significant reduction in IL-6 and IL-1β by cyanidin-3-glucose and cyanidin, respectively, was determined. Another important finding of this study is the capability of cyanidin to increase fractalkine mRNA expression, which could modulate microglia-neuron crosstalk, even under a pro-inflammatory (LPS-induced) environment [30].

Intracerebral hemorrhage (ICH) is an acute cerebrovascular disease without an effective medical treatment. However, prevention of secondary neural injury could be effectual to reduce nerve dysfunction. Increased numbers of M2 monocyte microparticles was reported in the plasma samples of ICH patients compared to M1 monocyte microparticles. However, there was no significant correlation between the increment of M2 monocyte microparticles and anti-inflammatory biomarkers/related cytokines. Reduced cellular activation or monocyte apoptosis was suspected as the result prior to further research [80]. On the other hand, Shi et al. (2016) revealed the efficacious effect of Sinomenine, an active alkaloid derived from *Sinomenium actum*, on ICH mitigation by modulating M1/M2 polarization. Besides enhancing M2 and attenuating M1 polarization, sinomenine also exhibited a similar capability in microglia-mediated neuron toxicity and apoptosis of neurons, as Cyanidin mentioned in a previous study. Furthermore, Sinomenine could effectively reduce microglia migration, infiltration, and activation in ICH, which could potentially protect neurons from the negative effect of activated microglia [35].

**Figure 4:**
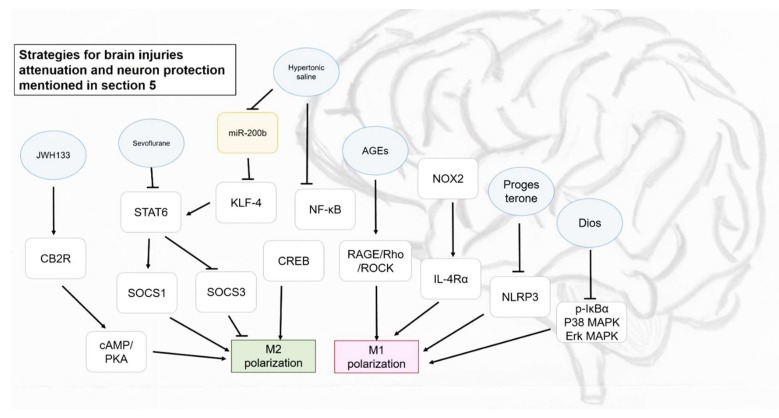
Some strategies proposed for brain injuries attenuation and neuron protection mentioned in Section 5. Blue circle, samples intervened; yellow block, microRNA intervened; white blocks, genes/proteins involved.

Besides pharmaceuticals, phytochemicals could have a positive impact on disease amelioration by modulating macrophage polarization as well. Surprisingly, some phytochemicals could also bring indirect but efficient protective effects on neurons from activated macrophages, providing therapeutic options in brain injury or neural damage. All of the potential strategies mediating M1 and M2 polarizations in brain diseases, which above-mentioned are as presented in Figure 4.

## 6. Role of Macrophages in Obesity and Metabolic Diseases

Obesity is usually accompanied by chronic and low-grade inflammation occurring in adipose tissue (AT). Similarly, macrophages in adipose tissue can significantly polarize into two classes of activated macrophages, as do other resident macrophages in other tissues. However, the polarization of M1 macrophages is often found to be causal when replacing M2 and therefore, the set-point of pro-inflammation is normally higher in obesity. On the other hand, obesity also takes part in the pathophysiology of most metabolic-related diseases, such as insulin resistance and diabetes, cardiovascular diseases, and cancers [59]. It is suggested that M2 macrophages prefer using fatty acid from β-oxidation as a fuel substrate, which indicates that targeting β-oxidation can be a strategy dealing with obesity and obesity-associated inflammatory disease [81]. Therefore, there are growing interests in clarifying the relationship between inflammation and obesity and metabolic diseases, and determining the possibility to ameliorate metabolic diseases via anti-inflammation action and M2 polarization enhancement.

The findings of Zhang [40] highlighted the therapeutic effect by remodeling homeostasis of macrophages. High fat diet (HFD)-induced obese mice were administrated with IL-4, polarized M_φ_ and M2 macrophages given intraperitoneally and through the tail veil, respectively. Although there was no difference in body weight between all of the experimental groups and HFD group, significant weight loss in inguinal adipose tissue (INAT), and decrement in INAT and epididymal adipose tissue (EAT) were observed in the M2 infusion group. M2 macrophages were found to increase in EAT and it was confirmed that the increment of M2 macrophages might be due to recruitment of infused macrophages but not the transformation of resident macrophages, with the homed M2 gradually decreasing to undetectable levels after three weeks. Furthermore, the diminution of M1 macrophage recruitment from blood after M2 infusion led to the attenuation of AT inflammation. Surprisingly, mRNA expression of thermogenic genes including *UCP1* (Uncoupling protein 1), *acox1*(Peroxisomal acyl-coenzyme A oxidase), *cidea* (Cell death activator CIDE-A), *prdm16* (PRdomain containing 16), and *cox8b* (Cytochrome C Oxidase Subunit 8B) was found to increase, implying a positive effect on thermogenesis up-regulation. Lastly, pro-inflammatory TNFα was reported to potently disrupt the insulin signaling cascade [81]. In this study, the M2 infusion group demonstrated amelioration in insulin resistance and significantly reduced lipid metabolism in triglycerides, high density lipoprotein, and low density lipoprotein cholesterol [40]. Hyperglycemia is described as a biochemical feature of diabetes, and unfortunately, high glucose levels have been reported to increase ROS-dependent pro-inflammatory activation. In other words, when compared to M2, M1 macrophages are more active under hyperglycemia conditions because they primarily use glucose as an energy substrate. Moganti et al. (2017) suggested that the differentiation of human monocyte-derived macrophages into M1 with significant production of TNFα was enhanced under hyperglycemic conditions in acute inflammatory phase but comparably alleviative in the chronic phase. Notably, the secretion of pro-inflammatory cytokines IL-1β, which are normally secreted by M1 macrophages in higher concentrations, had been found in M_φ_, M1, and M2 macrophages, indicating that hyperglycemia conditions could directly induce *IL*-*1β* expression. IL-1Ra and CCL18 are the cytokines that are produced by M2 macrophages, with the former counteracting the effect of pro-inflammatory effects of IL-1β, while the latter being a response to IL-4 at the late stage of differentiation. As expected, the level of IL-1Ra released by all M_φ_, M1, and M2 macrophages is a response to the expression of IL-1β mentioned above, while the expression of CCL18 was suppressed by hyperglycemia conditions. Elevation of IL-1Ra level was reported to be associated with an elevated risk of type II diabetes, prediabetes, and obesity, and the result showed that hyperglycemia could induce supportive M1/M2 related cytokines to accelerate the progression of diabetes [34]. Therefore, the regulation of M1/M2 polarization can be important to address the issue of obesity, diabetes, and insulin resistance amelioration.

The role of macrophages that are involved in pathogenesis of atherosclerosis is discussed in this section because one of the characteristics of atherosclerosis is the accumulation of lipid in tunica intima of medium- and large-sized arteries. The involvement and increment in Irgm1 levels were confirmed in both human atherosclerotic plaques and significantly elevated in a western diet-induced atherosclerosis model in *ApoE*^−/−^ mice when compared to wild-type mice. The result of co-localization of Irgm1 and M1/M2 markers showed a notably elevated level of M1 macrophage infiltration without any apparent difference in M2 markers. The effect of Irgm1 on M1 polarization was further confirmed by using *Irgm1*^+/−^ *ApoE*^−/−^ mice, which exhibited remarkably decreased atherosclerotic lesions after *Irgm* knockdown. Not only in Irgm1 haplodeficiency mice, *Irgm1*^+/+^ mice receiving *Irgm1*^+/−^ bone marrow also showed a dramatic reduction in atherosclerotic lesion and M1 marker expression. Lastly, M1-related transcription factor IRF5 significantly reduced in *Irgm1*^+/−^ mice but not *Irgm*^+/+^ and there was no effect on IRF4 (M2-related transcription factor), indicating that Irgm enhanced M1 but not M2 polarization [21]. Oxidation of low-density lipoproteins (LDLs) is directly related to the occurrence of atherosclerosis and these oxidized lipids can lead to formation of fatty streaks, one of the components in atherosclerotic plaques, via inflammatory signaling pathways. Expression of IFNα and IFNβ was dramatically induced by oxidized LDL, but not in other pro-inflammatory cytokines. Treating eosinophils with oxidized LDL markedly reduced IL-4 and IL-13 expression and induced expression of CD36, whereas CD36 was suggested as one of major components in the induction of IFN by oxidized LDL. Unsurprisingly, peritoneal macrophages treated conditioned medium of eosinophils with oxidized LDL treatment had significantly induced expression of the proinflammatory M1 marker, indicating the effect of activated eosinophils on M1 polarization enhancement via CD36 receptor signaling under a highly oxidized LDL concentration microenvironment [82]. Another study by Chen [17] proposed that curcumin possessed a preventive effect on atherosclerosis as a result of retuning cholesterol transport homeostasis and mediation of the inflammation response. Cholesterol uptake capacity of M1 macrophages was greater than M0 and internalization of cholesterol increased after being treated with higher curcumin concentrations. Meanwhile, cholesterol efflux mediated by either Apo-A1 or HDL was lower in M1 when compared to M0 and the effect could be elevated by curcumin treatment. Furthermore, although there was no effect on lowering lipid accumulation in M1, curcumin did enhance ox-LDL-stimulated foam cell formation with higher cholesterol ester accumulation. Furthermore, the effect of ox-LDL-induced inflammation was reversed by curcumin with a reduction in M1-related cytokine expression, including *IL*-*1β*, *IL*-*6,* and *TNFα*, which indicated that M1-macrophages played a role in dealing with cholesterol uptake and accumulation in the form of foam cells without secreting inflammatory cytokines after intervention with curcumin. Lastly, increments in CD36, ABCA1, and PPARγ levels were observed in ox-LDL-induced M1 macrophages, and it was confirmed that PPARγ and its downstream protein CD36 and ABCA1 were responsible for cholesterol uptake and efflux mediated by Apo-A1 [17]. Therefore, M1 macrophages exhibited a positive impact on dealing with cholesterol, which the ability enhanced by curcumin, and showed its anti-atherosclerosis potential.

Some research manifested that the intervention of some phytochemicals exhibits a pivotal role in normalizing or alleviating effects on inflammation related to obesity. Ko et al. (2014) used a transwell co-culture system of differentiated 3T3-L1 adipocytes and RAW264.7 macrophages to demonstrate the attenuation effect of theaflavin-3, 3′-digallate (TF3) on inflammation response activated by adipocytes. Differentiated 3T3-L1 dramatically activated macrophages to release inflammatory mediators, including nitric oxide (NO), TNFα, IL-1β, IL-6, and CCL2/MCP-1, and the effect was reversed after addition of TF3. Activation of M1 macrophages induced by adipocytes was confirmed with the expression of CD11c, which was dose-dependently reduced by supplementation of TF3, and the reduction in M1 macrophages was due to a phenotype switch from M1 to M2 (CD206^+^ cells). In addition, co-cultured M2 macrophages, which presented high levels of CD206, CD163, and arginase-1, with adipocyte being able to switch the M2 phenotype to CCR7 and CD86 (M1 markers), and again, the phenotype switch from M2 to M1 induced by adipocytes could be reversed by TF3. Intervention of TF3 in co-culture rendered inhibitory effects on phosphorylation of IKK, IκB, and p65, and reduced iNOS and COX-2 levels, indicating a repressive effect on M1 polarization. Besides this transwell system, conditioned medium was also used to confirm the paracrine interaction between two cell types. The results showed that both macrophages cultured in adipocyte- and macrophage-conditioned medium tend to release inflammatory cytokines and TF3 was suggested to interrupt paracrine interaction of both cell types. Moreover, although macrophages were reported to induce adipocyte hypertrophy, longer exposure of adipocytes to inflammatory cytokines may induce lipolysis and the release of free fatty acid, and these phenomena are the indicators of insulin resistance development in obesity by blocking insulin signaling cascades. Finally, it was concluded that TF3 could reduce cytokine release by adipocytes via the AMPK signaling pathway promoting phenotype shift of macrophages from M2 to M1 [28].

Another study of apigenin elucidated its effect on obesity-related inflammation improvement via the modulation of macrophage polarization. Apigenin efficaciously reduced infiltration of inflammatory cells in adipose tissue, and furthermore, dramatic reduction in pro-inflammatory cytokines and chemokine, CCL2 level in serum and adipose tissue was observed. Similar results were obtained in an *ob*/*ob* mice model with additional increment in IL-10 level. On the other hand, apigenin abolished the increased expression of M1 markers and up-regulated M2 polarization. Moreover, the polarization of adipose tissue macrophages was regulated so that mRNA of M1-related markers (CCL2 and CCL4) reduced, concomitant with enhancement in M2 markers (Arg1 and Ym1). The results of in vitro cell models and the *ob*/*ob* mouse model are in line with this study. Interestingly, because apigenin is known as a PPARγ ligand, the results also confirmed that regulation of macrophage polarization was through a PPARγ-dependent pathway. The mechanism revealed that apigenin could reduce the phosphorylation of IκBα and reverse the translocation of PPARγ/p65 complex into the nucleus, subsequently stopping the activation of NF-κB [23]. Similar to this study, Feng et al. (2014) suggested that the regulation of M1/M2 polarization could be achieved by chrysin (5, 7-di-OH-flavone) via PPARγ activation. Chrysin seemed to change M1/M2 status in peritoneal macrophages in obese mice, lower in M1, and higher in M2, with a lower infiltration of macrophages. The mediatory role of PPARγ in M1/M2 polarization was confirmed by using a PPARγ-specific antagonist and RAW264.7 that could overexpress PPARγ to ensure that the reduction of M1 and promotion of M2 transformation was through a PPARγ-dependent pathway [45].

Microenvironments can be critical for macrophage polarization. The concept of tissue stiffness was proposed and that micro-environment and environmental signals were important and being observed as correlating with morbidity of chronic inflammation [83]. Changes in stiffness of micro-environment may be affected by the activation of fibroblasts, proteins, activation of extracellular matrix (ECM), and crosslinking of tissues. The abovementioned cytokines released by differentiated adipocytes were responsible for polarization of macrophages, while cytokines from polarized macrophages at different states have a distinct impact on the adipocytes or adipose tissue as well, which indicates that the regulation of macrophage polarization can be an entry point for metabolic disease improvement.

## 7. Role of Macrophages in Joint Diseases and Hemodynamic Disease

As described above, besides resident macrophages in each tissue and organ, monocytes and M_φ_ macrophages are also broadly distributed in the blood circulation system throughout the body. Some injuries due to the involvement of bleeding or angiogenesis are reported to be highly related to inflammation.

Hemarthrosis is a disease related to joint disease and inflammation and leads to hemodynamic problems. FVIII-deficient mice were used to mimic hemarthrosis in hemophilia by introducing a 30-gauge needle in the right knee, mimicking single joint bleed with visible signs of bleeding as the outcome. Blood M1 macrophages in the hemarthrosis induction group significantly increased, and were highest in the first day of induction and gradually returned within seven days, while the M2 monocyte level sharply dropped, followed by gradual restoration and it reached the normal level on the seventh day. In comparison, there was no notable increment in M1 macrophages and the blood macrophages polarized from monocytes are majorly composed of M2 monocytes. Decrement in M2 and induction of M1 macrophage polarization after induction was observed in the red pulp of spleen, and the M1 monocytes sharply decreased on the first day of induction, followed by gradual recovery afterwards. Polarization of synovial macrophages and joint lavage into M1/M2 state were in a similar trend at the affected knee, but no significant effect was noted for the unaffected knee, as expected, which reached to the top on the first day and returned to the normal state within seven days [48]. Levels and changes of each polarized monocyte and macrophage were found to be distinct in circulating blood, spleen and synovial fluid after occurrence of hemarthrosis. Although there were only monocyte and macrophage transformation and a changing trend provided in this study, the information provided may still be useful if polarization of macrophage becomes a major strategy for therapeutic approaches for hemarthrosis in hemophilic patients. The effect of M1/M2 on joint diseases had also been discussed. It was reported that M1 significantly enhanced MMP-3 secretion in chondrocytes after co-culturing, meanwhile M2 induced the production of MMP-1 and ADAMTS5 expression, which played an important role in cartilage degradation or the progressive loss of bone in osteoarthritis [84].

Inflammation and immune dysfunction were reported to be responsible for placenta cell injury and it is highly related to hemodynamic disorder. On the other hand, there was a high relationship found between maternal low protein diet and negative impacts on offspring, including lower birth weight, rapid adipose tissue growth, increased risk of insulin resistance, and onset weight gain in the adult stage. Therefore, Vomhof-DeKrey et al. (2016) demonstrated the effect of maternal low protein diet on immune function and inflammatory response and birth weight. Up-regulation of the angiogenesis inducer, TNFα, was found in low protein diet placentas. However, reduction in M1 and elevation in M2 macrophages was determined in low protein diet placentas, which is in contradiction with the result of up-regulation of *TNFα* mRNA expression. Therefore, it was hypothesized that these macrophages with M2 markers might be intermediate M2-like macrophages, and not the typical M2a macrophages being discussed in most research. These M2 TNFα^+^ macrophages were found at higher levels in low protein diet placentas. On the other hand, invariant NKT cells, which can inhibit tumor angiogenesis via IFNγ, were significantly reduced in low when compared to normal protein diet placentas, with lower mRNA expression in *IFNγ* expression, indicating the occurrence of angiogenesis in low protein diet placenta, as evidenced by the greater density of CD31/PECAM (platelet endothelial cell adhesion molecule). Although maternal low protein diet could lead to angiogenesis, the compensatory angiogenesis did not contribute to the nutrition supply, but the opposite, placental dysfunction that is caused by inappropriate polarization may lead to fetal growth retardation and other negative effects aforementioned [50].

In brief, targeting M1/M2 macrophage transformation can be an effectual strategy to address several issues. Nonetheless, the inappropriate polarization of macrophages may still lead to unexpected or unwanted negative effects.

## 8. Role of Macrophages in Cancer Progression

There are several evidences showing the linkage between cancer and inflammation, for instance, the risk of developing many types of cancer increases with inflammatory diseases, but reduces after taking non-steroidal anti-inflammatory drugs, and the downstream of oncogenic mutations is also operated in the signaling pathways that are related to inflammation. Furthermore, cytokines and chemokines, as well as inflammatory cells, are found in the tumor microenvironment in both animal models and humans. Inflammation or infection is representative as extrinsic pathway that connects inflammation and cancer progression. Increasing evidences show that cancers share some key endogenous factors with inflammation, including transcription factors (NF-κB and STAT3 for instance), and inflammatory cytokines, including IL-1β, IL-6, IL-23, and TNFα. Among transcription factors, NF-κB is known as an important endogenous tumor promoter while STAT3 can increase tumor capacity in evading immune system by inhibiting maturation of dendritic cells [85]. Therefore, M1 macrophages polarization show a positive correlation to cancer progression. On the other hand, M2 macrophages are mainly recognized for balancing or counteracting the effect of M1 macrophages and promotes healing, tissue remodeling, and angiogenesis. However, it is believed that these characteristics may promote and contribute to tumor progression. M1-activated macrophages was reported to associate with hypoxic tissue microenvironment during acute infection rather than M2 macrophages. However, they behave differently in carcinoma, which M2-like phenotype is more likely to be present in hypoxic areas based on the signal deriving from microenvironment. These tumor-associated macrophages (TAMs) are reported to be responsible in senescence regulation, extracellular matrix remodeling, angiogenesis and lymphangiogenesis enhancement, cancer cell proliferation, invasion, and metastasis promotion. Moreover, M2-like macrophages that are exposed to stimuli, like IL-4, IL-10, and TGFβ, tend to express the migration-stimulating factor, the isoform of fibronectin [86]. Therefore, the role and function of tumor-associated macrophages must be clarified before taking therapeutic action.

A canine model was used to demonstrate the relation between tumor-associated macrophages and mammary tumors. All three tumor subtypes (1) Less aggressive tumors; (2) intermediate behavior tumors; and, (3) aggressive behavior tumors) were included in this study with around 30% having vascular invasion and 42% with nodal metastasis. It was suggested that there were higher macrophage counts in malignant tumors (group 2 and group 3 tumor histotype), rather than benign tumors, which supports the thesis that macrophages play a role in migration and invasion of mammary cancer cells. Furthermore, tumor subtypes with increased aggressiveness were detected having a higher count of tumor-associated macrophages. More abundant polarized M2 macrophages with the CD206 marker indicated the possibility that M2 macrophage infiltration in malignant mammary tumors might be related to tumor cell infiltration, motility, and vascular invasion. Meanwhile, M1 infiltration was found to be higher in benign tumor tissue, indicating phenotype shifting of macrophages from M1 to M2, which may lead to a pro-tumoral state or an environment that promotes cancer progression [47]. Similar results were mentioned by Juusola et al. (2018) that the serum from pancreatic cancer patients had significantly richer cytokines, and co-cultured monocytes and cancer cells in the serum of pancreatic cancer patients increased migration rate of cancer cells [87]. On the other hand, the idea of immunotherapy has been promoted in cancer treatments, by converting tumor-supportive macrophages into tumor-suppressive macrophages during tumor progression by regulating microenvironments. Microenvironment-responsive nanoparticles with IL-12 payload were used in order to re-educate macrophages locally from M2 into M1 in solid tumors without toxicity and overcame the problem of physical barrier in tumors [88]. Moreover, Oncostatin M (OSM), an inflammatory cytokine in the IL-6 superfamily, was suggested to be involved in M2 polarization via mTOR signaling complex 2 (mTORC2) and Akt, further promoting tumor growth and metastasis [89]. All of the findings above suggest that M2 has adverse roles and illustrates the importance of microenvironments during tumor progression.

Melanoma exosomes are naturally produced by melanoma cells and it is believed that tumor exosomes can lead to pathogenic process transmission. Endothelial cell GM-CSF was induced by melanoma exosome in pre-metastatic lymph nodes and GM-CSF could lead to the production of HIF-1α and HIF-2α in M1 and M2 polarized macrophages, respectively. Therefore, an imbalance in M1 and M2 macrophage polarization can result in blood vessel growth and tumor supportive neovasculature organization, respectively, whereas both of the outcomes lead to tumor growth [90]. Therefore, to achieve anti-tumor effects, not only the production of HIF-1α, HIF-2α, and VEGF should be inhibited, but also inappropriate macrophage polarization. On the other hand, it was pointed out that exosomal αvβ6 integrin played an important role in M2 polarization in prostate cancer and the αvβ6 integrin is actually shed by prostate cancer. In fact, peripheral blood mononuclear cells (PBMCs) that were isolated from β6-null mice were successfully transferred with β6, showing that β6 was not synthesized endogenously, and CD14^+^ human PBMCs were also successfully transferred with β6 with increased expression of CD163, indicating that the M2 polarization was due to the exosomal β6 shed from prostate cancer cells. To further confirm the result, down-regulation of αvβ6 in prostate cancer cells by using siRNA resulted in inhibition of M2 polarization, as expected. Furthermore, using monoclonal antibodies of αvβ6 could lead to blockage and resulted in accumulation of STAT1 and MX1/2. In the *Pten*^pc1/1^, tumor weight significantly reduced after injection of αvβ6 antibody, illustrating that the inhibition of αvβ6 could stop M2 polarization and result in anti-tumor effects [29]. Induction of M2 polarization promoted by miR-181a, which diminished the M1 phenotype when overexpressed, was reported by Bi et al. (2016). The presence of miR-181a was found to down-regulate KLF6 and C/EBPα in both mRNA and protein expressions via direct interaction [91].

So far, the polarization of macrophages into M2 state was reported having a negative impact on cancer attenuation. Therefore, unlike most circumstances in other diseases, M2 polarization inhibition has become a major strategy to retard cancer progression. β-elemene, an active component extracted from *Curcuma Wenyujin*, exhibited similar tumor-promoting effects in lung cancer by inhibiting migration, invasion transition of epithelial mesenchymal, concomitant with enhancing radiosensitivity of lung cancer cells [51]. Besides, Dong et al. (2017) demonstrated the effect of fenretinide, a kind of synthetic derivative of all-trans retinoic acid on the inhibition of M2 macrophage polarization in colon cancer. IL-4/IL-13 induced M2 polarization of BMDM with CD206, Fizz1, and PPARγ protein levels, which could be fully blocked by fenretinide without effect on M1 polarization. The inhibition of M2 polarization by fenretinide was due to blockage of STAT6 phosphorylation and the result was confirmed by using siRNA without further inhibition of STAT6 phosphorylation after intervention of siRNA. Conditioned medium of IL-4/IL-13 induced M2 polarized macrophages with/without fenretinide was used to evaluate the angiogenesis-promoting effect of M2 macrophages. Capillary-like structures were observed in the IL-4/IL-13-induced group and were abrogate in the fenretinide treatment group. In an APC^min/+^ transgenic mice model, tumorigenesis was inhibited with less CD206 marker being detected in tumor tissue without affecting body weight, showing that fenretinide could block tumor growth and this effect was not due to an improvement in obesity, but the abolishment of angiogenesis-promoting function of M2 macrophages [20]. Besides tumorigenesis, the invasiveness of cancer cells can also be affected by macrophages.

In summary, the roles of polarized macrophages at different states can bring uncertain effects in diseases. Therefore, an understanding of the roles of these macrophages and the mechanisms involved are compulsory prior to designing appropriate therapeutic strategies.

## 9. Conclusions

All of the studies analyzed in this paper pointed out the important roles of polarized macrophages in diseases that are strongly linked to immunological conditions. There is no fixed role of macrophages in different diseases, with one presenting therapeutic roles in the first condition possibly playing a deteriorative role in another, and the significance of each polarized macrophage at every stage in disease progression also potentially differing. Therefore, controversial results may be suggested in different studies that are discussing the same diseases because conformation is intricate, intriguing and integrated differently by various factors. Furthermore, macrophages activated by different stimuli may lead to various cytokines’ production and the composition of cytokines may change microenvironments, while the effect can also be inversed, from resident tissue to microenvironment that finally reaches the macrophages. A plethora of studies and reviews are imperative to cover as many of the circumstances which may occur when the living environment becomes more complicated. Not only synthetic drugs and pharmaceuticals, but also phytochemicals/nutraceuticals, may also possess compelling effects for health improvements. More efforts are required for clarifying all the states, roles, and mechanisms of macrophage polarization, perhaps with phytochemicals or drugs for more efficacious results, and the impact of microenvironments of different compositions may also facilitate therapeutic actions. Eventually, understanding macrophage regulation may lead to be of help in medication development.

## Figures and Tables

**Table 1:** Inducers and samples intervened in isolated macrophages and/or macrophage cell lines polarization with genes expressed, cytokines/chemokines produced, and surface markers being observed and used as indicators of polarized macrophages of different states in some recent studies. (Samples intervened were underlined to differentiate with inducers.)

Isolated Macrophages and/or Macrophage Cell Lines	Inducers/Samples	Gene Expressed	Cytokines/Chemokines	Surface Markers	References
RAW264.7	LPS + IFNγ	*IL*-*1β*, *IL*-*6*, *TNF*-*α*, *iNOS, CCL2* (M1) *Arg1*, *Ym1*, *IL*-*4*, *Mrc*-*1*, *Mgl1* (M2)	IL-1β, IL-6, TNF-α (M1) -	-	[15]
RAW264.7	LPS (200 ng/mL)	-	TNF-α, IL-6, GM-CSF, IL-1α, G-CSF (M1), COX-2 (M1) -	CD86 (M1) CD206 (M2)	[16]
RAW264.7	LPS (100 ng/mL) + IFNγ (2.5 ng/mL)	*IL*-*1*, *IL*-*6*, *TNF*-*α* (M1) -	-	CD86 (M1)	[17]
CD14^+^ isolated human peripheral blood monocytes	M-CSF/IFNγ (100 ng/mL) + M-CSF LPS (100 ng/mL)	*IL*-*6* (M1) *IL*-*10* (M2)	TNFα, IL-6 (M1) IL-10 (M2)	-	[18]
Peritoneal macrophage	LPS (20 ng/mL) + IFNγ (1 µg/mL)/IL-4 (20 ng/mL)	*IL*-*1β*, *IL*-*6*, *TNFα*, *IL*-*12*, *iNOS* (M1) *IL*-*10*, *Fizz*-*1* (M2)	- IL-10, Fizz-1, Ym-1 (M2)	-	[19]
RAW264.7	IL-4/IL13 (10 ng/mL) + Fenretinide	*Fizz*-*1*, *PPARγ* (M2)	STAT6 (M2)	CD206 (M2)	[20]
Peritoneal macrophage	LPS (100 ng/mL) + IFNγ (10 ng/mL)/IL-4 (200 ng/mL)	*Irf5*, *iNOS* (M1) *Fizz*-*1*, *Irf4* (M2)	-	-	[21]
RAW264.7 ANA-1 THP-1 Murine peritoneal cells	LPS (500 ng/ mL)/IL-4 (10 ng/mL) + curcumin	*CCL3*, *IL*-*1β* (M1) *Arg*-*1*, *Ym1*, *CD206* (M2)	MHCII, CCR7, TNF-α, (M1) MGL1/2 (M2)	CD80 (M1)-	[17]
RAW264.7 Bone marrow derived macrophage	LPS (100 ng/mL) + IL-4 (10 ng/mL)/10 ng/mL + Pentamethoxyflavanone	*IL*-*1β*, *IL*-*6*, *TNF*-*α*, *iNOS* (M1) *Fizz*-*1*, *Ym*-*1*, *CD206* (M2)	-	CD11c (M1) CD206 (M2)	[22]
Murine peritoneal Macrophages RAW264.7	LPS/IL-4 + Apegenin	*CCR2*, *TNF*-*α*, *CCL3*, *CCL4*, *IL*-*1β*, *NOS2* (M1) *Fizz*-*1*, *Ym*-*1*, *Arg*-*1*, *MGL1*/*2*, *CD163*, *CD206*, *IL*-*1r* (M2)	TNF-α, CCL2, IL-1β, IL-6, IL-12, CCR7, MHCII (M1) IL-10, Arg-1, MGL1/2 (M2)	CD80 (M1) CD206 (M2)	[23]
RAW264.7	Curcumin (25 µmol/mL)	*IL*-*4*, *IL*-*13*, *MMR*, *Arg*-*1* (M2)	IL-4,IL-13, MMR and Arg-1 (M2)	-	[24]
Murine peritoneal macrophages	LPS (100 ng/mL)	-	iNOS, COX-2 (M1)	-	[25]
RAW264.7	Tim-3 overexpressing	*TNFα*, *IL*-*12*, *IFNγ*, *IL*-*17* (M1) *IL*-*10* (M2)	-	-	[26]
Mouse peritoneal macrophages	LPS (10-100 ng/mL) + ginsenoside Rg_3_	*COX*-*2*, *iNOS*, *IL*-*1β*, *TNF*-*α* (M1) *Arg*-*1*, *IL*-*10*, *TGF*-*β*, *TNFα*, *Ym1* (M2)	-	-	[27]
RAW264.7	LPS/Adipocytes-conditioned medium + Theaflavin-3, 3′-digallate	*iNOS*, *TNF*-*α*, *IL*-*6*, *IL*-*1β*, *COX*-*2* (M1) *Arg*-*1*, *PPARγ* (M2)	TNF-α, IL-1β, IL-6, MCP1, CCR7 (M1) IL-10, STAT3 (M2)	CD11c, CD86 (M1) CD206, CD163 (M2)	[28]
Human and murine blood monocyte	Exosomal αvβ6	-	CCL2/CCR2, STAT3 (M2)	CD204, CD163 (M2)	[29]
Microglia cell line N9	LPS (0.1 µg/mL)/IL-4 (20 ng/mL) + Anthocyanins	*IL*-*6*, *iNOS*, *IL*-*1β* (M1) *Ym*-*1*, *CCR*-*2*, *Arg*-*1*, *CX3CR1* (M2)	-	-	[30]
Primary microglia cells	IL-4 (10 ng/mL) + sevoflurane	*Arg*-*1*, *Ym*-*1*, *IL*-*10* (M2)	-	-	[31]
Primary Kuffer cells Bone Marrow-derived macrophage (BMDM)	IL-4	*TNF*-*α*, *NOS2* (M1) *Arg*-*1*, *IL*-*10*, *Retnlα* (M2)	-	CD80 (M1) CD206, CD163 (M2)	[32]
Bone Marrow-derived macrophage (BMDM) treated mice (liver tissue)	LPS + IFNγ/IL-4	*iNOS*, *IL*-*12*, *TNFα* (M1) *Arg*-*1*, *Mrc*-*1*, *Ym*-*1* (M2)	CCL2, CCL3 (M1) Lyc6 (New classification)	-	[33]
CD14+ peripheral blood mononuclear cells	IFNγ (100 ng/mL)/IL-4 (10 ng/mL) + M-CSF (5 ng/mL)	-	TNF-α, IL-1β IL-1Rα, CCL18 (M1)	-	[34]
Primary microglia cells	Erythrocyte lysates + Sinomenine	*iNOS*, *IL*-*1β*, *TNF*-*α* (M1) *IL*-*10*, *Arg*-*1* (M2)	-	-	[35]
BV2 microglia cells Primary microglia cell culture	LPS (200 ng/mL) + IFNγ (20 ng/mL)/IL-4 (10 ng/mL)/SSRI	*IL*-*1β*, *IL*-*6*, *iNOS*, *TNFα* (M1) *IL*-*10* (M2)	IL-1β, TNF-α (M1) IL-10 (M2)	CD86 (M1) CD206 (M2)	[36]
RAW264.7 Bone Marrow-derived macrophage (BMDM)	LPS (10 ng/mL) + IFNγ (10 ng/mL)/ IL-4 (10 ng/mL) + isomeranzin	*IL*-*1β*, *IL*-*6*, *TNF*-*α* (M1 Raw 264.7) *IL*-*1β*, *IL*-*6*, *iNOS*, *TNF*-*α* (M1 BMDM) *Arg*-*1*, *Fizz*-*1*, *Ym*-*1* (M2)	IL-1β, IL-6, TNF-α (M1) -	CD11, iNOS (M1) -	[37]
Primary microglia cells BV-2 cells	LPS (0.1-1 µg/mL) + Diogenin glucoside	*iNOS*, *TNF*-*α*, *IL*-*6*, *IL*-*23*, *IL*-*17*, *IL*-*1β* (M1) *IL*-*10*, *IL*-*1Rα*, *G*-*CSF*, *SOC3*, *COX*-*1*, *COX*-*2*, *Arg*-*1*, *Fizz*-*1*, *Ym*-*1*, *Chi3l3* (M2)	TNF-α, IL-6 (M1) IL-10, IL-1Rα (M2)	CD206 (M2)	[38]
RAW264.7	LPS (500 ng/mL) + IFNγ (20 ng/mL)	-	-	CD86 (M1) CD206 (M2)	[39]
Bone Marrow-derived macrophage (BMDM)	-	-	Arg-1 (M2)	CD206 (M2)	[40]
Peripheral blood CD14+ monocyte	GM-CSF (50 ng/mL)+LPS (1 ng/mL) + IFNγ (20 ng/mL)/M-CSF (100 ng/mL) + IL-4 (20 ng/mL) + pentacyclic tirterpene Lupeol	*IL*-*1β*, *IL*-*12*, *TNF*-*α* (M1) *IL*-*10* (M2)	-	CD86 (M1)CD206 (M2)	[41]
Mouse peritoneal macrophages	LPS (1 µg/mL) + baicalin	*TNFα*,*IL*-*23* (M1) *IL*-*10*, *Arg*-*1* (M2)	- Fizz-1 (M2)	-	[42]

Arg1, Arginase 1; CCL, C-C motif chemokine ligand; CCR, C-C chemokine receptor; Chi3l3, Chil3 chitinase-like 3; COX-2, cyclooxygenase-2; CX3CR1, CX3C chemokine receptor 1; Fizz-1, resistin like alpha; GM-CSF, Granulocyte-macrophage colony-stimulating factor; IFNγ, Interferon gamma; iNOS, Inducible nitric oxide synthase; IRF, interferon regulatory factor; LPS, lipopolysaccharides; M-CSF, macrophage colony-stimulating factor; Mgl1, C-type lectin domain family 10, member A; Mrc1, Macrophage mannose receptor-1; NOS2, Nitric oxide synthase 2; PPARγ, Peroxisome proliferator-activated receptor gamma; Retnlα, Resistin-like molecule alpha; SOCS3, Suppressor of cytokine signaling 3; Tim-3, T-cell immunoglobulin and mucin-domain containing-3; TGF-β, Transforming growth factor beta; TNF-α, Tumor necrosis factor alpha; Ym1, Chitinase 3-like 3.

**Table 2:** Inflammation inducers and samples intervened in macrophages polarization in/isolated from various tissues with gene expressed, cytokines/chemokines produced and surface markers being observed and used as indicators of polarized macrophages of different states in some recent studies. (Samples intervened were underlined to differentiate with inducers.)

	Inducers/Samples	Gene Expressed	Cytokines	Surface Markers	References
Ipsilateral and contralateral hippocampi	CCl	*IL*-*1β*, *IL*-*6*, *TNF*-*α*, *IFN*-*γ* (M1) *IL*-*10*, *IL*-*13*, *Arg*-*1*, *Fizz*-*1*, *Mrc*-*1*, *Ym*-*1* (M2)	IL-1β, IL-6, TNF-α, IFN-γ (M1) IL-4, IL-10, IL-13 (M2)	-	[43]
Brain tissue	Cuprizone + Progesterone	*iNOS*, *MHC II*, *TNF*-*α* (M1) *Trem*-*2*, *Arg*-*1*, *TGF*-*β* (M2)	IL-18 (M1)-	CD86 (M1) CD206 (M2)	[44]
Colonic tissue Peritoneal macrophage	DSS + LZ205 Alum + LZ205	*IL*-*1β*, *IL*-*6*, *TNF*-*α*, *CCL2*, *iNOS* (M1)	IL-1β, IL-6, TNF-α, MPO, iNOS (M1) -	-	[15]
Tumor region at colonic tissue	APC^min/+ mice^ given high fat diet + Fenretinide	-	-	CD206 (M2)	[20]
Arteriosclerotic plaque	Irgm1^−/−^ and ApoE^−/−^ given western diet	-	iNOS (M1) Arg-1, Fizz-1 (M2)	CD16/32 (M1)-	[21]
Adipose tissue	High fat diet + Chrysin	-	IL-1β, TNF-α (M1) IL-10 (M2)	NOS2 (M1)-	[45]
Serum, BALF and Lung tissue	LPS/ Puncture (CLP) surgery + Pentamethoxyflavanone	*IL*-*1β*, *IL*-*6*, *TNF*-*α* (M1)-	IL-1β, IL-6, TNF-α (M1)-	CD11c (M1)-	[22]
Adipose tissue	High fat diet + Apigenin	*CCL3*, *CCL4* (M1) *Ym*-*1*, *Arg*-*1* (M2)	TNF-α, CCL2, IL-1β, IL-6, IL-12, CCR7, MHCII (M1) IL-10, MGL1/2, Arg-1 (M2)	CD80 (M1) CD206 (M2)	[23]
Myocardial tissue in heart injury	EAM (treatment) + Curcumin	*IL*-*1β*, *iNOS* (M1) *MMR*, *Arg*-*1* (M2)	-	-	[24]
Liver tissue	TAA	-	IFN-γ, TNF-α, MCP-1, Iba-1, MHC II (M1) IL-4, TGF-β, Gal-3, Hsp25 (M2)	CD68 (M1) CD163, CD204 (M2)	[46]
Colonic tissue	TNBS + 4-methoxychocarpin	*TNF*, *IL*-*1β*, *IL*-*17A*, *Arg*-*2* (M1) *IL*-*10* (M2)	-	CD86 (M1)-	[25]
Intestinal macrophages isolated from intestinal lamina propria cells	DSS + Tim-3 (protein)	*TNF*, *IL*-*1β*, *NOS2*, *IL*-*6*, *IL*-*12* (M1) *Arg*-*1* (M2)	-	CD16/32 (M1) Dectin-1 (M2)	[26]
Benign and malignant tumor (breast cancer)	Canine mammary tumors	-	NOS2 (M1)	-CD206 (M2)	[47]
Blood monocyte Red pulp macrophage and monocyte in murine spleen Synovial tissue Joint lavage	Hemathrosis induction	-	MHC1, MHC2, Ly-6C (M1)	CD86 (M1) CD163 (M2)	[48]
Microglia cells isolated from myeloid cells	Alcohol	-	MHC II, iNOS (M1) MMR (M2)	CD86 CD16/32 (M1) CD206 (M2)	[49]
Perihematomal region of cerebral tissues	Blood injection into striatum	-	iNOS, IL-1β, TNF-α (M1)	-	[35]
Placenta cells Splenocytes	Maternal low protein diet	*IL*-*12*, *IL*-*6*, *IL*-*10*, *IL*-*1β*, *NOS2* (M1) *Mrc1(CD206)*, *Mgl1(CD301)* (M1)	TNF-α (M1) IL-6, TNF-α (M2b), CCL4 (M2)	CD274 (M1) CD163 (M2)	[50]
Liver tissue/serum/BALF Colonic tissue	LPS/DSS/TNBS + isomeranzin	*IL*-*1β*, *IL*-*6*, *TNF*-*α*, *iNOS* (M1)	IL-1β, IL-6, TNF-α (M1)	-	[37]
Colonic tissue	DSS + C.EDA	-	IL-1β, IL-6, TNF-α, IL-10 (M1)	CD11c (M1) CD206 (M2)	[39]
Colonic tissue	DSS + pentacyclic triterpene Lupeol	*IL*-*12*, *IL*-*6*, *IL*-*1β*, *TNF*-*α*, *iNOS* (M1)-		CD86 (M1) CD206 (M2)	[41]
Mouse colonic lamina propria mononuclear cells	DSS + baicalin	*TNF*-*α*,*IL*-*23* (M1) *TNF*-*α*, *IL*-*23*, *IL*-*10*, *Arg*-*1* (M2)	-	-	[42]

Arg1, Arginase 1;BALF, Bronchoalveolar lavage fluid; CCL, C-C motif chemokine ligand; CCR, C-C chemokine receptor; Chi3l3, Chil3 chitinase-like 3; COX-2, cyclooxygenase-2; CX3CR1, CX3C chemokine receptor 1; DSS, Dioctyl sodium sulfosuccinate; Fizz-1, resistin like alpha; GAL-3, Galectin-3; GM-CSF, Granulocyte-macrophage colony-stimulating factor; Iba-1, Ionized calcium-binding adapter molecule 1; IFNγ, Interferon gamma; iNOS, Inducible nitric oxide synthase; IRF, interferon regulatory factor; HSP25, Heat shock protein 25; LPS, lipopolysaccharides; Ly6c, lymphocute antigen 6 complex; M-CSF, macrophage colony-stimulating factor; MCP1, Monocyte chemoattractant protein 1; Mgl1, C-type lectin domain family 10, member A; MHCII, Major hiscompatibility complex II; Mrc1, Macrophage mannose receptor-1; MPO, Myeloperoxidase; NOS2, Nitric oxide synthase 2; PPARγ, Peroxisome proliferator-activated receptor gamma; Retnlα, Resistin-like molecule alpha; SOCS3, Suppressor of cytokine signaling 3; TAA, Thioacetamide; Tim-3, T-cell immunoglobulin and mucin-domain containing-3; TGF-β, Transforming growth factor beta; TNBS, 2, 4, 6-Trinitrobenzenesulfonic acid solution; TNF-α, Tumor necrosis factor alpha; Tream2, Triggering receptor expressed on myeloid cells 2; Ym1, Chitinase 3-like 3.
